# Design and Production of a New FeCoNiCrAlCu High-Entropy Alloy: Influence of Powder Production Method on Sintering

**DOI:** 10.3390/ma14154342

**Published:** 2021-08-03

**Authors:** Eduardo Reverte, Monique Calvo-Dahlborg, Ulf Dahlborg, Monica Campos, Paula Alvaredo, Pablo Martin-Rodriguez, Elena Gordo, Juan Cornide

**Affiliations:** 1 Department of Materials Science and Engineering, IAAB, University Carlos III of Madrid, Av. de la Universidad, 30, 28911 Leganés, Spain; campos@ing.uc3m.es (M.C.); palvared@ing.uc3m.es (P.A.); 100420728@alumnos.uc3m.es (P.M.-R.); egordo@ing.uc3m.es (E.G.); jcornide@ing.uc3m.es (J.C.); 2 GPM CNRS-UMR6634, University of Rouen Normandie, 76801 St-Etienne-du-Rouvray, France; monique.calvo-dahlborg@univ-rouen.fr (M.C.-D.); ulf.dahlborg@univ-rouen.fr (U.D.)

**Keywords:** powder metallurgy, high entropy alloy, atomisation, microstructure, X-ray diffraction, Thermo-Calc, alloy design, differential thermal analysis

## Abstract

The structure of FeCoNiCrAl1.8Cu0.5 high-entropy alloys (HEA) obtained by two different routes has been studied. The selection of the composition has followed the Hume–Rothery approach in terms of number of itinerant electrons (e/a) and average atomic radius to control the formation of specific phases. The alloys were obtained either from a mixture of elemental powders or from gas-atomised powders, being consolidated in both cases by uniaxial pressing and vacuum sintering at temperatures of 1200 °C and 1300 °C. The characterization performed in the sintered samples from both types of powder includes scanning electron microscopy, X-ray diffraction, differential thermal analysis, and density measurements. It was found that the powder production techniques give similar phases content. However, the sintering at 1300 °C destroys the achieved phase stability of the samples. The phases identified by all techniques and confirmed by Thermo-Calc calculations are the following: a major Co-Ni-Al-rich (P1) BCC phase, which stays stable after 1300 °C sintering and homogenising TT treatments; a complex Cr-Fe-rich (P2) B2 type phase, which transforms into a sigma phase after the 1300 °C sintering and homogenising TT treatments; and a very minor Al-Cu-rich (P3) FCC phase, which also transforms into Domain II and Domain III phases during the heating at 1300 °C and homogenising TT treatments.

## 1. Introduction

The design and development of high-entropy alloys (HEAs) differ from traditional alloys systems where several secondary elements are added to one or two principal ones, such as in iron-based, aluminium-based or titanium-based alloys. Thus, HEAs have emerged as a new type of functional and structural metallic materials with the idea of multi-principal elements in an equimolar or near equimolar ratio to maximise the configurational entropy (ΔS_config_) of a solid-solution phase [1,2]. In most cases, HEAs form simple crystalline structures of body-centred cubic (BCC) or face-centred cubic (FCC) types but also, less commonly, of hexagonal close-packed (HCP) type [3,4,5,6], thereby supressing in some cases the formation of intermetallic compounds with different properties due to the synergy between the constituent elements [7].

Recently, a classification of HEAs was proposed using the average value of the number of itinerant electrons (e/a) in the alloy and the average atomic radius [8,9]. The importance of e/a to understand and predict the phases in solid solutions was stressed as early as 1966 [10]. In [11], the use of e/a is discussed for the determination of crystalline Hume–Rothery phases as opposed to the standard approach using the total number of valence electrons (VEC) (which includes the d-electrons). By applying this approach for HEAs as solid solutions, a classification and design methodology was proposed in [8,9]. From the variation of the average magnetic moment per atom and the hardness as a function of e/a and the atomic radius, three domains could be identified: one domain (e/a < 1.53) containing alloys with cubic close-packed (FCC) structure, a second one (1.53 < e/a < 1.88) containing alloys with mixed and/or complex structures, and a third one (e/a > 1.88) containing alloys with structures belonging to the body-centred cubic (BCC) family.

Typical production methods for high-entropy alloys follow the casting route [7,12,13] via arc or induction melting. However, the present investigation focuses on the development of a HEA by powder metallurgy (PM) due to the high and flexible potential of PM to design multi-component materials. Two routes were used for the powder production: elemental powder blending and gas atomisation. The powder was further processed by uniaxial pressing and sintering as a proof of concept. The common PM production method for HEAs is mechanical alloying followed by Spark Plasma Sintering (SPS) as the preferred consolidation method [14]. However, no way to fabricate a HEA with optimised physical properties for specific technological application using this production route has, until now, been found. The two methods of HEA production discussed in this study have only been used in about 25% of the publications about PM-HEAs to date, while nearly 75% have been focused on mechanical alloying [12,15,16].

Following the approach presented in [8,9], a PM BCC high-entropy alloy, CrCoFeNiAl_1.8_Cu_0.5_, has been designed and processed, with a focus on properties suitable for high-temperature applications. Alloys based on the equiatomic CrCoFeNi composition have been studied widely. Moreover, additions of Al to this alloy, which has a simple FCC structure, have been shown to induce the formation of BCC structures [17], leading to a considerable increase in yield strength. Hence, the Al content was fixed with a ratio of 1.8. Furthermore, the addition of Cu has been found to affect the mechanical behaviour [18], but it was reduced up to a molar ratio of 0.5, due to previous reported segregation on microstructure [19]. From these fixed Al and Cu molar ratios, the e/a and radius values, 1.89 and 1.337 respectively, make the designed alloy to belong to Domain III (e/a > 1.88), with BCC-type structures [8,9].

In the present study, the e/a-designed HEA was produced by a PM route (pressing and sintering) from powders produced by two techniques: atomisation and blending elemental powders. The starting powder morphology, particle size distribution, densities, and chemical homogeneity were studied. In addition, the structural differences between the sintered samples obtained from the two types of powders were analysed in detail by differential thermal analysis and X-ray diffraction.

## 2. Materials and Methods

**Figure 1:**
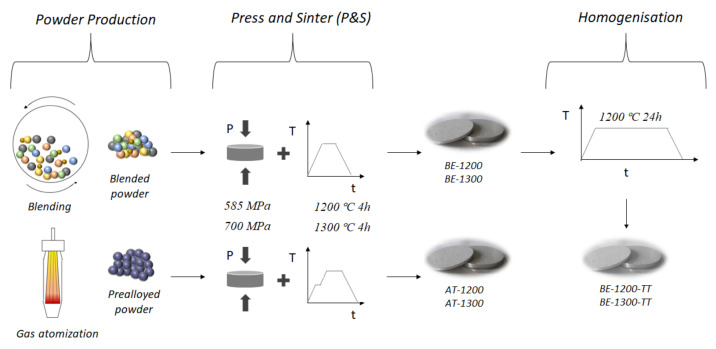
Graphical scheme of the processing routes.

The production of powders with a nominal composition CrCoFeNiAl_1.8_Cu_0.5_ has followed two different routes, as described in Figure 1: (1) gas atomisation and (2) blending elemental; the powders were consolidated by subsequent uniaxial pressing and vacuum sintering (P&S) in a high-vacuum tube furnace, model Carbolite-Hut 15/50/450 (Carbolite, Derbyshire, UK). For the first route of the gas atomisation process, the raw metals (over 99% purity) were melted in an induction furnace, poured, and atomised in a gas atomiser from Atomising Systems Limited, Sheffield, UK. The gas atomisation was performed with an injection of nitrogen at 12 bar pressure, and the temperature of the molten HEA was 1600 °C, measured by the introduction of a thermocouple just before starting the pouring.

The second route to obtain the HEA was by using all elemental blended powders. The starting elemental powders were Ni (average particle of 4 µm, Skyspring Nanomaterials, Houston, TX, USA), Fe (average particle of 3 µm, H.C. Starck, Munich, Germany), Cr (average particle of 5 μm, Skyspring Nanomaterials, Houston, TX, USA), Al (average particle of 15 μm, Skyspring Nanomaterials, Houston, TX, USA), Co (average particle of 1.6 µm, Alfa Aesar, Kandel, Germany), and finally Cu (35 µm of average particle size, as-atomised). The powders were blended in a Turbula shaker mixer (WAB, Muttenz, Switzerland) for 1 h. The particle size of the powders was measured by Mastersizer 2000 from Malvern Instruments Ltd., Worcestershire, UK. In addition, the interstitial elements of the powders were studied by analysing the oxygen/nitrogen and carbon/sulphur contents in a LECO TC-500 and LECO TC CS-200 (both from LECO, St Joseph, MI, USA), respectively.

Both routes were processed by conventional pressing and sintering (P&S) in Ø16 mm disks. The atomised powder was mixed with 3 wt.% Acrawax to improve the compressibility and pressed in a double effect uniaxial die at 585 MPa. The route from blended powders did not need Acrawax, as the powders showed a great compressibility performance at 700 MPa on the same press. In the rest of this text, the acronyms for the samples from the atomised and the blended powders routes will be “At-” and “BE-”, respectively.

Finally, the sintering was performed in high vacuum (10^−5^ mbar) with a heating rate of 5 °C/min up to 500 °C, in the case of the atomised powder when an isothermal dwell of 30 min was maintained to ensure the total removal of the wax, then heated up to 1200 °C or 1300 °C for 4 h to densify the materials. In order to sinter the blended powders, no isothermal dwell was needed, and it was heated up directly to 1200 °C or 1300 °C.

These two sintering cycles were selected from the DTA curve of the blended powders. The lack of pressure or high heating/cooling rates during consolidation, like in fast sintering techniques, is compensated with the high sintering temperature, below the melting of the powder, to facilitate the densification of the alloy.

After sintering, the samples from the blended route were homogenised by a heat treatment at 1200 °C for 24 h in vacuum (10^−2^ mbar). In order to identify each technique, a summary and scheme of the parameters and routes followed are shown in Table 1 and Figure 1.

The thermal characterisation was performed by differential thermal analysis (DTA) before and after the sintering process using “Setaram Setsys Evolution” equipment (Setaram Instrumentation, Caluire-et-Cuire, France). The DTA was conducted under argon atmosphere up to 1600 °C in alumina crucibles with a heating and cooling rate of 10 °C/min.

The microstructural and morphology observations of the sintered samples and the atomised powder was made by scanning electron microscopy (SEM) in a FE-SEM FEI Teneo (Thermo Fisher, Hilsboro, OR, USA) equipped by energy-dispersive X-ray spectroscopy (EDS) detector. The accuracy, precision, and detection limits of energy-dispersive X-ray spectroscopy vary due to multiple sources of errors, with a reasonable 5% of relative error for major components [20].

The X-ray diffraction (XRD) analysis was performed in a Siemens D5000 diffractometer (Siemens, München, Germany) with a Cu cathode ray tube (λ = 1.5406, 1.5444 Å) on the grinded surface of each sample. The 2θ angle range studied starts at 20° and it goes to a maximum of 120°.

Thermo-Calc 2017a [21] software (Thermo-Calc Software, Stockholm, Sweden) with SOL5 database was used to obtain the phase equilibrium diagram for the CrCoFeNiAl_1.8_Cu_0.5_ composition.

## 3. Results and Discussion

The particle size distribution and the impurities content of the atomised powders are shown in Table 2. These D-values are related to the cumulative volume distribution, more precisely to the undersize D-value in which an *x* volume percentage (10%, 50%, and 90%) of the particles are smaller than the stated size. The atomised powder shows a D_50_ value of approximately 50 µm and an D_90_ upper limit of 93 µm. The content of impurity elements present in the atomised powder was also measured due to their influence on mechanical properties [22,23]. From the results, it can be confirmed that there was not important oxidation or nitriding effect during the atomisation. Finally, the powder density was measured by He pycnometer giving the value of 6.96 ± 0.02 g/cm^3^. By using the elemental densities of each element in the composition, the theoretical density of a solid solution can be estimated by the rule of mixtures (ROM) in Equation (1), typically used in composite materials and metal alloys [24,25,26,27]. However, the calculation is stated as an approximation due to the mismatch between crystal structures of the elements and their respective individual densities:

(1) $$\rho_{alloy}=\overset{n}{\underset{i}{\sum }}c_{i}M_{i}/\overset{n}{\underset{i}{\sum }}(c_{i}M_{i}/\rho_{i})$$

where $c_{i}$, $M_{i}$, and $\rho_{i}$ are the atomic concentration, the atomic weight, and the density of the ith element, respectively. Nevertheless, the calculation by this equation gives a density of 6.43 g/cm^3^, far below the atomised powder density of 6.96 g/cm^3^. This disparity of densities between experimental and theoretical data has been found also in some previous reports [28,29]. Furthermore, the unit cell density of the atomised powder has been calculated based on the lattice parameter of the BCC structure measured by XRD in Equation (2):

(2) $$\rho_{unit\;cell}=(\overset{n}{\underset{i}{\sum }}c_{i}\cdot N\cdot M_{i})/(N_{a}\cdot a^{3})$$

where *N*, $N_{a}$, and a are the number of atoms in the unit cell, the Avogadro’s number, and the lattice parameter, respectively. In this case, the density expected from Equation (2) is 6.79 g/cm^3^, closer to the 6.96 g/cm^3^ obtained experimentally from the atomised powder. This density mismatch could be related to a presence of a minor FCC, since Equation (2) only takes account of BCC structures.

**Figure 2:**
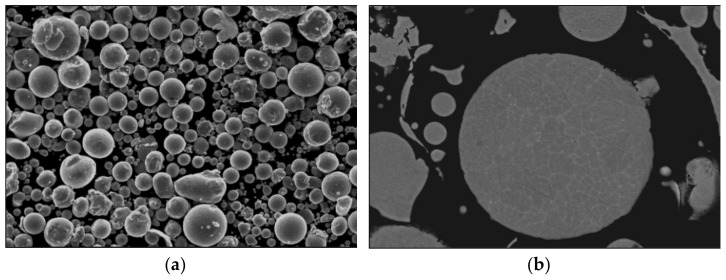
SEM images from the atomised powder (**a**) morphology of the powders (SE mode) (**b**) transverse section of atomised powder particles (BSE mode).

The morphology and compositional analyses made by SEM-EDS are shown in Figure 2 and Table 3, respectively. The large majority of the powder particles are spherical in shape, as well as the very few satellites. The compositional analysis of the EDS shows results close to the nominal composition of the alloy. The highest difference is observed in iron and aluminium content, probably related to minor aluminium losses during the melting and pouring process prior to the gas atomisation [28].

**Figure 3:**
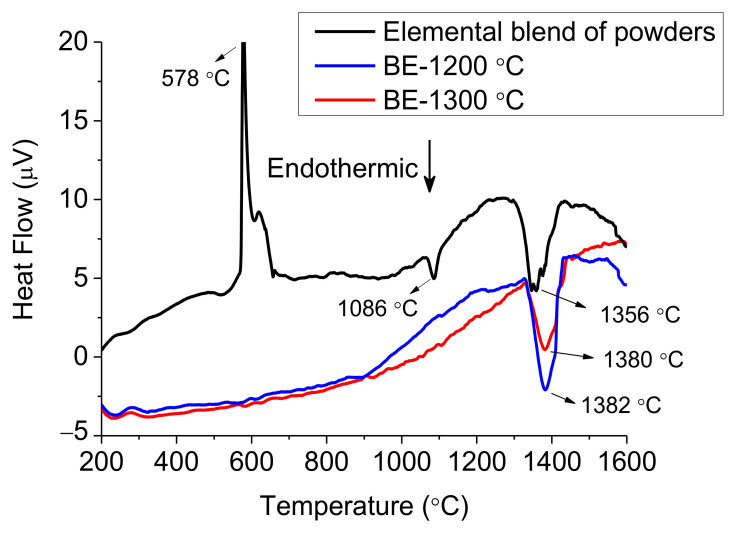
Differential Thermal Analysis (DTA) on heating of blended powders and BE-samples.

**Figure 4:**
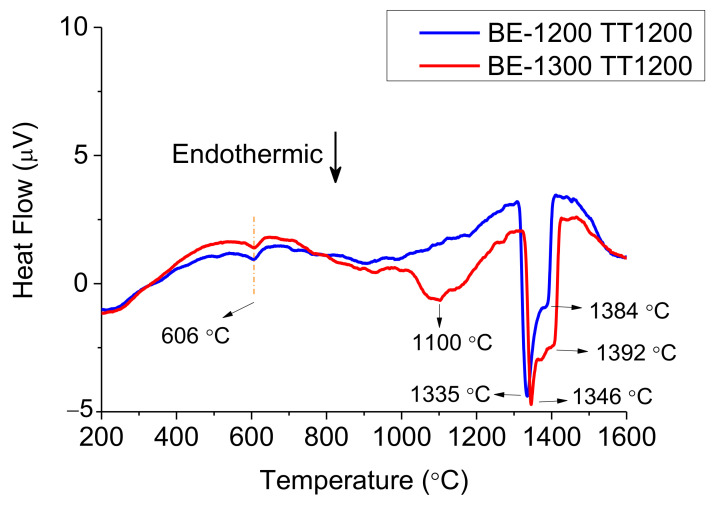
Differential Thermal Analysis (DTA) on heating of as-homogenised samples (TT-samples).

**Figure 5:**
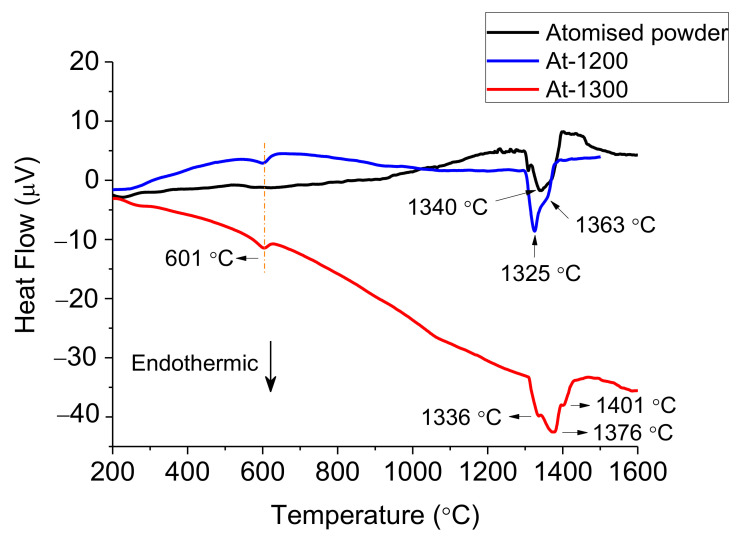
Differential Thermal Analysis (DTA) on heating of atomised powders and At-samples.

The thermal behaviour on heating of each starting powder alongside with each sintered alloy was analysed by DTA, as presented in Figure 3, Figure 4 and Figure 5. The melting temperatures would indicate the optimal sintering temperature of the alloys. Moreover, phase transformations play an important role to understand the possible phases on each microstructure.

The DTA results from the blended powders in Figure 3 show one exothermic reaction and two endothermic transformations. The lowest temperature reaction is identified as a two-stage exothermic reaction at 578 °C that could indicate the formation of Al-Cu-Fe phases as concluded in [30]. However, the melting of the aluminium powder is still expected to occur around these temperatures in the mixture, and hence, the exothermic reaction and the endothermic melting are probably combined within the 570–660 °C temperature range when the exothermic reaction occurs. At higher temperatures, the appearance of an endothermic peak at 1086 °C suggests the melting of Cu and the subsequent enrichment of these elements in the blended powders. Finally, a wide endothermic peak at 1356 °C appears, which is attributed to the melting range of the elemental powders, but not Al and Cu. Regarding the thermal results from BE-samples presented in Figure 3, both curves only show a melting reaction between 1380 °C and 1382 °C with an onset of 1328 °C, higher than the blended powder. Moreover, these two peaks have similar peak width, suggesting comparable phases compositions.

The thermal analysis of the as-homogenised samples shown in Figure 4 presents the heating events around 600 °C with a little endothermic peak for both BE-1200TT and BE-1300TT that could represent the dissolution of an ordered FCC-L12 phase as previous authors have found [31]. Around 1100 °C for BE-1300TT, a change in the slope suggests a phase transformation. It also reveals a multi-step melting process with an onset temperature of 1330 °C, similar to the BE-samples.

The curve for the gas atomised powder in Figure 5 exhibits some irregularities in the temperature range around 1250 °C before reaching a two-stage melting reaction around 1340 °C, which suggests the appearance of various phases. In the DTA heating curves for the At-samples, both curves present two endothermic peaks, the first one again at 601 °C that could correspond also to the dissolution of an ordered FCC-L12 [31] and the second one that corresponds to the melting. Moreover, around 1100 °C there is a change of slope in the curve for At-1300 samples suggesting a phase transformation. The melting appears above 1300 °C in a two-stage reaction for At-1200 and a three-stage reaction for the At-1300, suggesting the dissolution of multiple phases.

It is to be noted that the DTA curve for BE-1200TT and At-1200, on one side, and BE-1300TT and At-1300, on the other side, present similar series of events. In particular, the at-1300 and the BE-1300TT present an additional transformation around 1100 °C (see Figure 4 and Figure 5).

All these features in thermal behaviour indicate a diversity in microstructural phases or compositions and highlight the importance of the thermal treatment during the powder production method.

The density of atomised powder was set as a reference since no hollow or porous particles were detected (Figure 2b). Therefore, the densities of sintered materials, shown in Table 4, were calculated using 6.96 g/cm^3^ as the theoretical density. According to the results, the highest densification occurs at 1300 °C for the At-samples. This is in accordance with earlier measurements, where it has been shown that higher sintering temperatures lead to denser samples [32]. However, in the case of BE-samples, BE-1200 alloy shows higher relative density than the BE-1300. These results suggest a disparity between microstructures and lattice parameters in the crystalline structure apart from the porosity factor.

**Figure 6:**
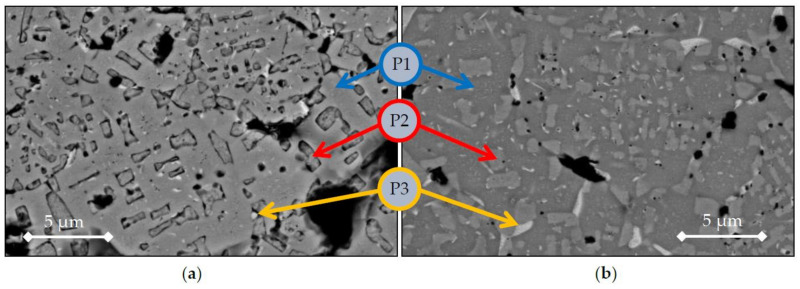
SEM images from samples sintered from elemental powder, (**a**) BE-1200, (**b**) BE-1300, and details of P1, P2, and P3 phases described in Table 4 and Table 5.

Figure 6 shows the microstructures of BE-samples from elemental powders. Both cycles display similar phases: one main phase that can be considered as a matrix (Figure 6, P1), a second polygonal-shape phase (Figure 6-P2), and a residual bright phase (Figure 6, P3), which can be seen from the small bright spots in the microstructure. Table 5 presents the EDX composition measured for each BE-sample with each e/a calculation and fitted domain from [9,33], that is, Domain I: e/a < 1.53, Domain II; 1.53 < e/a < 1.88, Domain III: e/a > 1.88. Two similar columns are introduced in Table 6 and Table 7. The analysis of the phases reveals a high concentration of Co, Ni, and Al in the matrix (phase P1), exceeding the nominal composition, whereas the polygonal shape seems to have a high concentration of Cr and Fe (phase P2). Lastly, the residual bright phase is considered as a Cu-rich phase, phase P3. From the e/a values of each phase, the P1 phase (Co-Ni-Al) seems to belong to Domain III, related to BCC types of structure. Phase P2 seems to belong to Domain II, and Phase P3 to Domain I after 1200 °C and Domain II after 1300 °C sintering heating.

These findings will be compared with XRD results in the following section of the text. However, it is to be stressed that for phases P2 and P3, which are in small amounts, the error bar on the determination of the composition by EDS can be large. Hence, the very different e/a values for Al and Cu, 3 and 1 respectively, can lead to very different biased Domains. These precipitates are rare in terms of volume fraction, so they could not be detected in XRD nor in DTA. The difference in density is also noted in SEM observations of the transversal sections, where BE-1200 samples have higher porosity than BE-1300 samples.

**Figure 7:**
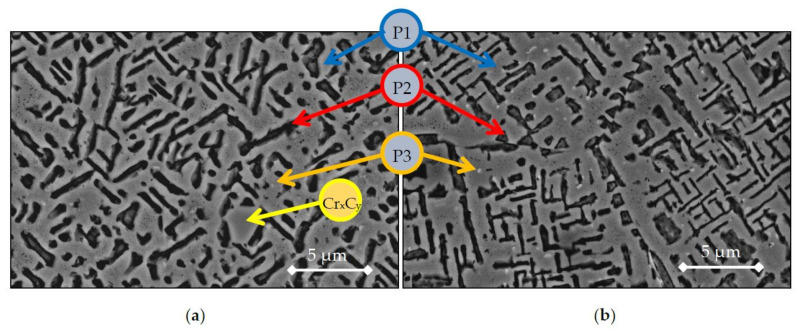
SEM images of samples sintered from atomised powder (**a**) At-1200 (**b**) At-1300.

The microstructures of At-samples sintered from atomised powder are shown in Figure 7. There are several differences by changing the blended powders as starting material compared to the prealloyed powder. The microstructure shows the presence of a grey matrix with an elongated and crisscrossed phase probably induced by a spinodal decomposition during the sintering process. This crisscrossed phase appears as a dark phase due to the etchant used to highlight the microstructure in the metallographic preparation. Moreover, it is also visible the presence of chromium carbides due to the contamination from the wax used as lubricant at the pressing stage. The EDX compositions and e/a values and Domain of the phases are presented in Table 6. Both phases follow similar values as previous microstructures; one main phase (P1) Co-Ni-Al-rich that belongs to the domain III, a second crisscrossed phase (P2) Cr-Fe-rich in domain II. However, the third minor phase (P3) is in Domain III for both 1200 °C and 1300 °C. The term Cu-rich (P3) is related to those phases showing an increase of an element compared to the composition of the as atomised powder (shown in Table 3).

**Figure 8:**
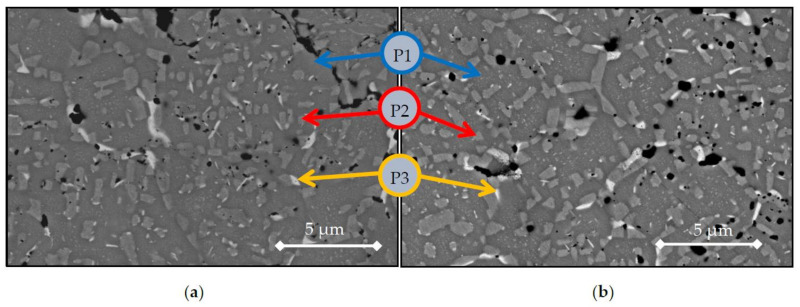
SEM images of as-homogenised samples (**a**) BE-1200 (**b**) BE-1300 in BSE mode.

Finally, the microstructures and compositions of the heat treated samples are shown in Figure 8 and Table 7. The influence of the homogenisation treatment on the phases will be discussed later as a function of the sintering treatment performed before on the powders, i.e., 1200 °C or 1300 °C.

**Figure 9:**
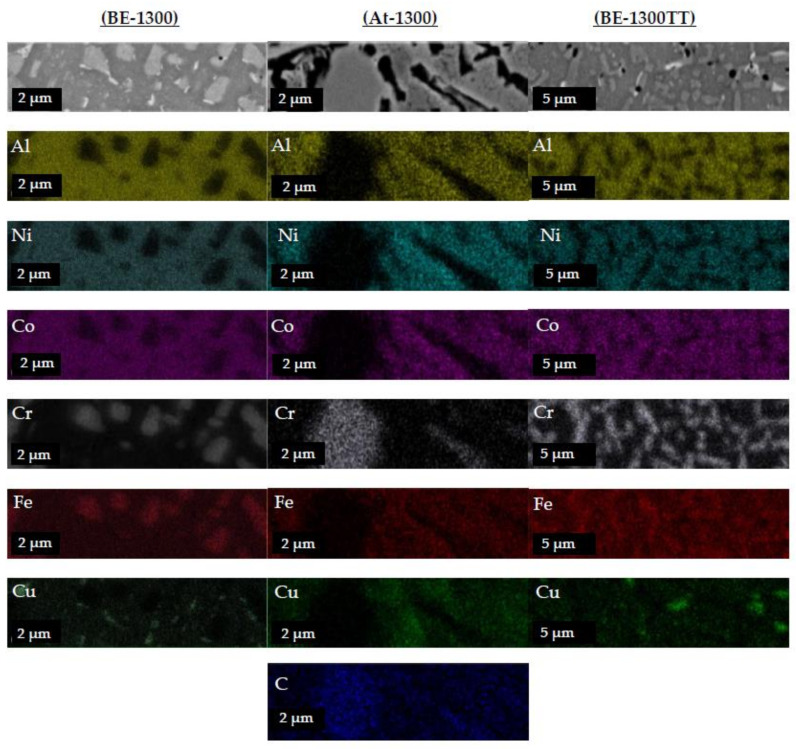
EDS area mappings performed on CrCoFeNiAl_1.8_Cu_0.5_ HEA by processing method.

Furthermore, a compositional mapping along the microstructures on the phases of the alloys sintered at 1300 °C is shown in Figure 9. In the compositional mappings, there are three different regions where the elements are located preferentially. These regions are visible in heat-treated and BE-samples, similar to those observed in SEM images previously: the main Co-Ni-Al rich phase (P1) as a matrix of the microstructure, a polygonal-shape Cr-Fe rich phase (P2), and copper segregation (P3). On the heat-treated samples, the microstructures from BE-1300TT are effectively finer and more homogenised than BE-1300.

However, in At-samples, the crisscrossed Fe-Cr phase is not distinguishable due to the etchant. During metallographic preparation, the etchant preferentially dissolved the Fe-Cr phase, leaving only the chromium carbides as contamination embedded in the Co-Ni-Al matrix and the copper segregation.

**Figure 10:**
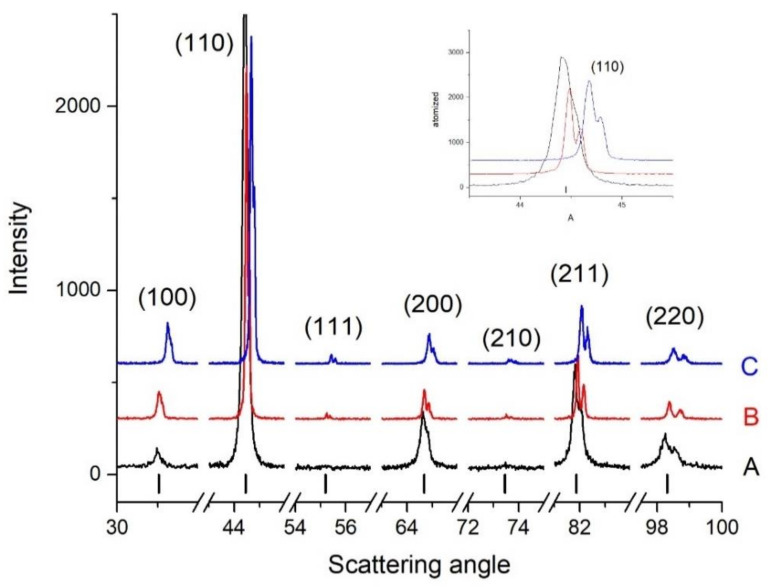
XRD patterns of (A) the atomised powder, (B) BE-1200, and (C) BE-1300. The indices and the vertical bars indicate the positions of the diffraction peaks for an ordered bcc (B2) structure.

In Figure 10, the XRD patterns of the atomised powder and the BE-samples are compared. The main phase in the three samples is a BCC phase which corresponds to P1 and Domain III in both Table 5 and Table 6. In atomised powder (A) and BE-1200 alloy (B), the structure is slightly distorted BCC (BCT) with lattice parameters 2.8814(2) and 2.8785(2) Å, respectively. These structures are also reported by previous authors when the Al content exceed the equimolar ratio [34,35]. Some additional peaks indicate the presence of a B2 phase, in smaller amount, which could be the Phase 2 (P2) in Table 5 and Table 6. The diffraction pattern from the BE-1300 alloy (C) looks very similar to the BE-1200. However, the structure is the same but the BCC lattice is found to be more distorted with BCT lattice constants a = 2.8753(8) and c = 2.860(2) A. A small difference in structure between the two sintered samples is expected though, from the DTA curves shown in Figure 3. It is to be noted that both the Kα1 and the Kα2 contributions are well separated in all patterns, indicating that no or very small size broadening is present. Thus, all diffracting regions are larger than around 1 µm. Phase 3 (P3) is reported to belong to Domain I at 1200 °C and II at 1300 °C from Table 5 and Domain III from Table 6. However, the very small amount detected in the microstructures in Figure 6, Figure 7 and Figure 8 might be the reason that it is not detected in Figure 10.

**Figure 11:**
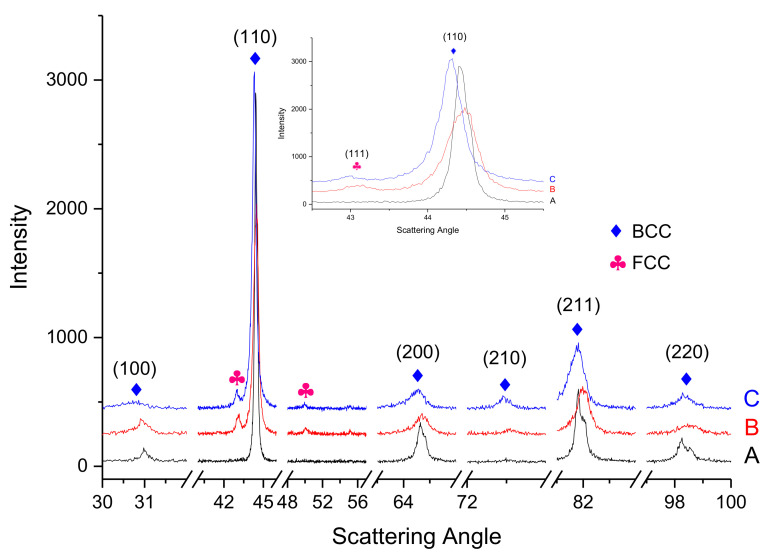
XRD patterns of the (A) atomised powder, (B) At-1200, and (C) At-1300.

The diffraction patterns of the At-alloys from atomised powder in Figure 11 also indicates the appearance of a BCC-type structure (Phase P1) with some ordered regions, a B2/BCC phase (P2). However, a shoulder in the (110) peak might correspond to a (111) peak of an FCC structure and suggests a possible appearance of another phase with a lattice parameter of 3.64(2) Å. Depending on the error bar applied on the concentration values for P3 in Table 6, the attributed Domain is III, due to the e/a values of Al and Cu. A possible interpretation of both this uncertainty and the two peaks by the main one in Figure 11 is that there are two additional phases in very small amounts: one of FCC-type and an unidentified 4th one, one of the two containing C.

**Figure 12:**
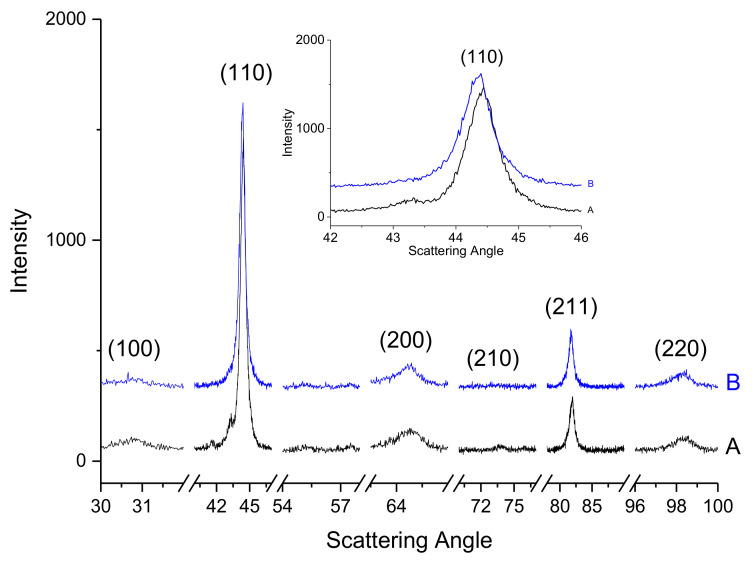
XRD patterns of as-homogenised (A) BE-1200TT and (B) BE-1300TT.

Similar results were found in the as-homogenised samples in Figure 12. The main phase is still of BCC type (P1). However, a reduction of the intensity of the (100) peak of the ordered phase could indicate its partial dissolution or rearrangement of the atoms to a more disordered one. Although of less statistics than in Figure 10 and Figure 11, it can be concluded that phase P2 has transformed to an FeCr-sigma phase. Additionally, an FCC phase might be present but visible mainly on the 1200 °C sintered sample rather than the 1300 °C one.

**Figure 13:**
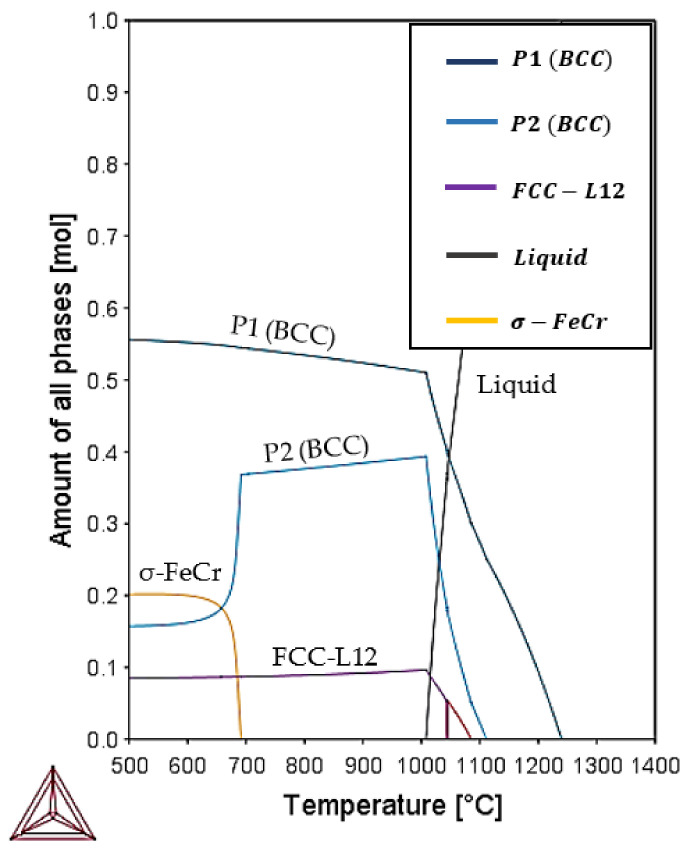
Theorical prediction of microstructural phases over temperature by Thermo-Calc.

Finally, a thermodynamic calculation of the HEA made by Thermo-Calc software^®^ using the SOL5 database [21] is presented in Figure 13.

The difference in phases between the atomised powder and sintered HEAs relies mainly on the cooling rate and the contrast between the concepts of melting and solid-state sintering in the production methods. The atomised powder comes from the fast solidification (≈10^5^ K/s) [36] of the molten material, which leads to an almost single phase, and a major homogeneous distribution of the elements. On the other hand, the sintering does not reach a liquid phase, and the HEA formation is controlled by the diffusion of the different elements under slow heating and cooling rates (≈10 K/min). Then, the phases observed in Thermo-Calc are more similar to the as-sintered conditions, as these are closer to the equilibrium conditions, compared to the as-atomised powders [36,37].

The theoretical predictions by Thermo-Calc shown in Figure 13 represent the mol percent of stable phases over temperatures up to 2000 °C. The BCC phases seen in XRD observations are confirmed to be thermodynamically stable on the graph. In addition, the melting reaction of the alloy is stated at 1240 °C; this temperature varies from the melting of 1281 °C calculated using the rule of mixtures (ROM) in Equation (3) [38] and the temperatures around 1380 °C measured by DTA of the BE-samples.

(3) $$Tm=\overset{n}{\underset{i=1}{\sum }}c_{i}{\left(Tm\right)}_{i}$$

where (*Tm*)i is the melting point of the ith component of the alloy and c_i_ is the mole percent of each alloy component.

Although the ROM method may only represent a rough estimation for alloy parameters, also used normally for density prediction, it shows the difficulty of approximating high-entropy alloy basic properties in contrast to classic alloy design with one main element.

Despite the good agreement between the phases obtained by Thermo-Calc and the observed by experimental techniques, there is a remarkable difference between the melting point obtained by Thermo-Calc and the onset temperature in all the DTAs. This disagreement between the result Figure 13 and the DTA curves shown in Figure 3, Figure 4 and Figure 5 in that the temperature region in which solid and liquid phases coexist are more than one hundred degrees apart. The rates used in DTA are very slow, and it can be assumed that this has no significant effect on the temperature regions in which all alloys melt and solidify. Thus, the Thermo-Calc calculation is not accurate in this respect, while the predicted phase contents seem to be more reasonable.

The calculations in Figure 13 also present the formation of a minor FCC-L12 phase, in accordance with a possible dissolution of an ordered FCC-L12 found on DTA curves and referenced by [31].

Finally, the Thermocalc graph also shows the formation of σ-FeCr at temperatures below 700 °C.

The Thermo-Calc calculations allow us to provide explanations for the following previous observations:

(1) The 1200 °C temperature is within the domain of appearance of the BCC phase and below the melting temperature in DTA. This can explain why the 1200 °C samples, At-1200 and BE-1200, are similar and closer to expectation.
(2) When heated up to 1300 °C, a possible transformation to sigma phase for the P2 phase can appear.
(3) The phases expected from the e/a calculations in Table 5 and Table 6 are confirmed.

The HEA composition has demonstrated a large variety of phases and atomic structures depending on the powder process technique:

- P1 ordered phase (Co-Ni-Al) dominates in all routes with a BCC structure, in accordance with the e/a value in domain III and the XRD results.
- P2 phase also appears in smaller amount with B2 structure. This phase seems to transform to an FeCr-sigma phase when the 1300 °C sintering is applied. These findings agree with the Thermo-Calc calculation and the e/a values (Domain II). The DTA analysis also shows multi-stage melting reactions of these phases. The possibility of sigma phase formation was considered through the process and yet, the characteristic patterns from the tetragonal structure, high peaks approximately at 42° and 47°, were not found in any sample in the XRD data due to the high number of peaks and the low intensity and resolution. These sigma phases are typically formed by binary phase pairs like Fe-Cr or Co-Cr [39].
- P3 phase, Cu-rich, as an FCC type was too minor to be detected. The Domain III calculated for this phase in At-1200 samples could be due to the presence of C inside or to a 4th phase.

## 4. Conclusions

The production of a high-entropy alloy was addressed by two processing routes: elemental powder blending and gas atomisation. After sintering, the evolution towards two different microstructures and atomic structures was verified by SEM and XRD.

The calorimetric, microstructural, diffraction, and Thermo-Calc investigations confirm the e/a calculations of the different phases: a major BCC (P1), a B2 phase (P2) in smaller amount, and a very minor FCC (P3) phase.

The 1200 °C sintering process is to be preferred to the 1300 °C one, as the latter induces the transformation of the secondary phase to sigma phase.

Although very attractive, the production route giving At-samples results in the formation of carbides or carbon-containing phase. The Carbon contamination comes from the wax used as lubricant at the pressing stage.

This route of production from atomised powder results in microstructures free from secondary phases, although the formation of a small fraction of chromium carbides is unavoidable when lubricants are used for sample consolidation by pressing.

The homogenisation treatment TT is probably at too high a. temperature, as it seems to result also in the transformation of P2 to a sigma phase.

This disparity between methods clearly illustrates the importance of the production parameters to fabricate a high entropy alloy. Moreover, in the design process of HEAs, the e/a approach has shown its suitability for the prediction of the crystallographic structures for the HEA, which is useful to identify potential alloy compositions of interest, especially considering that the results obtained by this method are consistent to the final structures obtained by both production routes (blended and prealloyed), a BCC crystalline structure.

## Figures and Tables

**Table 1:** Summary of processing conditions applied on sintered and homogenised samples.

**Starting Powder**	**Nomenclature**	**Sintering Temperature (°C)**	**Sintering Time (h)**
Elemental Powder	BE-1200	1200	10
	BE-1300	1300	4
Atomised pre-alloyed powder	At-1200	1200	4
	At-1300	1300	4
**Heat Treatment**	**Nomenclature**	**Homogenisation Temperature (°C)**	**Homogenisation Time (h)**
Homogenised samples sintered from elemental powder	BE-1200TT	1200	24
	BE-1300TT	1200	24

**Table 2:** Characterisation measurements of the atomised powder: density, particle size, and interstitial contents.

**Pycnometer Density (g/cm^3^)**		6.96 ± 0.02
**Particle Size**	**D10 (µm)**	13.5
	**D50 (µm)**	50
	**D90 (µm)**	93.5
**% Oxygen**		0.026 ± 0.001
**% Nitrogen**		0.018 ± 0.001
**% Carbon**		0.04 ± 0.01
**% Sulphur**		0.004 ± 0.002

**Table 3:** EDS composition analysis of the atomised powder.

	Al	Fe	Cr	Co	Ni	Cu
Atomised powder (at%)	25 ± 3	20 ± 1	14.8 ± 0.7	16 ± 1	15.7 ± 0.3	7.4 ± 0.4
Nominal composition (at%)	28.5	15.8	15.8	15.8	15.8	7.9

**Table 4:** Relative density of the sintered samples.

Sample Type	Pycnometer Relative Density (%)
BE-1200	93.2 ± 0.1
BE-1300	82.6 ± 0.4
At-1200	93.8 ± 0.1
At-1300	97.7 ± 0.1

**Table 5:** EDS compositional analysis (at%) of phases detected on as-sintered samples from blended powders.

	Processing Type	Cr	Co	Fe	Ni	Al	Cu	e/a *	Domain *
Co-Ni-Al rich phase (P1)	BE-1200	5	**19**	12	**21**	**35**	8	2.01	III
	BE-1300	7	**18**	13	**20**	**34**	7	1.98	III
Cr-Fe rich phase (P2)	BE-1200	**30**	14	**22**	11	18	4	1.71	II
	BE-1300	**38**	12	**26**	7	13	3	1.63	II
Cu-rich phase (P3)	BE-1200	6	4	5	4	10	**68**	1.26	I
	BE-1300	8	9	8	8	20	**46**	1.56	II
Theoretical composition	-	15.8	15.8	15.8	15.8	28.5	7.9	1.89	III

* e/a prediction of the phase domain from [9,33].

**Table 6:** EDS compositional analysis (at%) of phases detected on as-sintered samples from atomised powders.

	Processing Type	Cr	Co	Fe	Ni	Al	Cu	e/a *	Domain *
Co-Ni-Al rich phase (P1)	At-1200	9	**14**	7	**16**	**48**	5	2.16	III
	At-1300	4	**19**	15	**22**	**29**	9	1.9	III
Cr-Fe rich phase (P2)	At-1200	**28**	14	**31**	9	13	5	1.71	II
	At-1300	**27**	13	**30**	8	11	11	1.65	II
Cu-rich phase (P3)	At-1200	14	12	8	15	29	**22**	1.78	III
	At-1300	3	11	10	16	24	**36**	1.69	III
Theoretical composition	-	15.8	15.8	15.8	15.8	28.5	7.9	1.89	III

* e/a prediction of the phase domain from [9,33].

**Table 7:** EDS compositional analysis (at%) of phases detected on as-homogenised samples.

	Heat Treatment	Cr	Co	Fe	Ni	Al	Cu	e/a *	Domain *
Co-Ni-Al rich phase (P1)	BE-1200TT	5	**19**	13	**22**	**33**	8	1.98	III
	BE-1300TT	6	**16**	12	**19**	**39**	8	2.06	III
Cr-Fe rich phase (P2)	BE-1200TT	**36**	14	**28**	8	12	2	1.66	II
	BE-1300TT	**38**	12	**25**	8	12	5	1.61	II
Cu-rich phase (P3)	BE-1200TT	3	4	3	4	14	**71**	1.34	I
	BE-1300TT	12	9	5	12	30	**32**	1.74	II
Theoretical composition	-	15.8	15.8	15.8	15.8	28.5	7.9	1.89	III

* e/a prediction of the phase domain from [9,33].

## Data Availability

The data presented in this study are available on request from the corresponding author.
